# Spectroscopic, Thermodynamic and Molecular Docking Studies on Molecular Mechanisms of Drug Binding to Proteins

**DOI:** 10.3390/molecules27238405

**Published:** 2022-12-01

**Authors:** Tanveer A. Wani, Seema Zargar, Afzal Hussain

**Affiliations:** 1 Department of Pharmaceutical Chemistry, College of Pharmacy, King Saud University, P.O. Box 2457, Riyadh 11451, Saudi Arabia; 2 Department of Biochemistry, College of Science, King Saud University, P.O. Box 22452, Riyadh 11451, Saudi Arabia; 3 Department of Pharmacognosy, College of Pharmacy, King Saud University, P.O. Box 2457, Riyadh 11451, Saudi Arabia

## 1. Introduction

Molecular recognition, which is the process of biological macromolecules interacting with each other or various small molecules with a high specificity and affinity to form a specific complex, constitutes the basis of all processes in living organisms. Proteins, an important class of biological macromolecules, realize their functions through binding to themselves or other molecules. Protein–ligand interactions play an important role in most biological processes, such as signal transduction, cell regulation, and immune response. Therefore, the study of protein–ligand interactions continues to be very important in life science fields. Since the recognition of their importance at the beginning of the 20th century, investigations into binding parameters have received significant attention, and understanding of these interactions is therefore central to understanding biology at the molecular level. Moreover, knowledge of the mechanisms responsible for protein–ligand recognition and binding also facilitates the discovery, design, and development of drugs.

Quantifying the binding of chemical entities to a protein is an important early screening step during drug discovery and is of fundamental interest for estimating safety margins during drug development. Current progress in experimental and computational methods for identifying and characterizing ligand binding sites on protein targets has provided biological insights that are significant for drug discovery. In addition, because the aim of rational drug design is to make use of knowledge of the structural data and protein–ligand binding mechanisms to optimize the process of finding new drugs, an in-depth understanding of the nature of molecular recognition/interactions is also of great importance. For a deeper understanding of the molecular recognition between a protein and its ligand, physicochemical mechanisms underlying the protein–ligand interaction, the binding kinetics, the basic thermodynamic concepts and relationships relevant to protein–ligand binding, and the driving forces/factors of binding and enthalpy–entropy compensation are investigated using experimental and theoretical methods and using various techniques such as isothermal titration calorimetry (ITC), surface plasmon resonance (SPR), and fluorescence, circular dichroism, and UV absorption spectroscopy.

## 2. Contributions

Fourteen research papers covering various aspects of drug development that help in understanding an interaction of various ligands to proteins were published in this Special Issue. These studies investigated the molecular mechanisms and structural changes in target proteins involved in the interaction with various viruses and bacteria. In addition, drug–protein interactions, drug–drug interaction mechanisms, anti-glycating and antioxidant activity, binding affinity, network-pharmacology-driven investigations, and targeted anticancer treatments were also investigated.

Proteins are essential biological macromolecules and have enormous importance in all physiochemical process. The native or folded forms of proteins are necessary to perform their biological functions. Pharmacologically profiling drugs offers an understanding of the interactions of vital therapeutic drugs or their derivatives with either plasma or target tissue proteins. In medicinal chemistry, studies pertaining to plasma proteins and drug binding are attracting researchers across the globe because these studies provide a platform to study drugs’ behaviors and actions, thereby delineating their transport and distribution characteristics in the circulatory system. The simultaneous administration of two or more drugs might lead to competition between the two drugs to bind to a similar site. The conformation of the protein binding pockets can be altered on interaction with ligands, thereby influencing the binding of other ligands to the same binding pockets. These studies will help in understanding the various binding mechanisms involved in the interaction between various ligands and proteins.

Respiratory syncytial virus (RSV) is a lower respiratory tract pathogen that causes pneumonia and bronchiolitis in children, especially those younger than five years of age. There is no licensed vaccine or any therapeutics available against RSV. The only available preventive measure is the injection of a monoclonal antibodies cocktail (palivizumab) specific to the fusion glycoprotein, which may decrease the severity of disease and rate of hospitalization in infants. In the their study, Hamza et al. [1] examined the structural changes in ectodomain G protein (edG) in a wide pH range. The absorbance results revealed that protein maintains its tertiary structure at physiological and highly acidic and alkaline pH. Fluorescence quenching, molecular docking, and MD simulation studies suggested strong binding between the edG and heparan sulfate. The study suggested that heparan-sulfate-mimicking compounds can be used to target the effective host–pathogen interaction.

Oxidative stress is an imbalance between ROS production and antioxidant defense, which causes cell damage at high levels. The ROS and their pathophysiological effects depend on the concentration, type, and specific production site. When ROS are at a high level, they react with DNA, proteins, cell membranes, and other molecules, causing cellular damage and producing other more reactive radicals. Zargar and Wani [2] investigated protection given by quercetin against CCl4-induced neurotoxicity in rats. In addition to various biochemical parameters, the authors also used VirtualToxLab to study the interaction between quercetin and various proteins to understand the molecular basis of the protective potential of quercetin against carbon tetrachloride toxicity on rat brains. The authors also hypothesized a possible mechanism for quercetin protection against CCl4 toxicity in the rat brain.

Human serum albumin (HSA) is a major transporter and the most abundant protein in the plasma. It exhibits ligand-binding capabilities, acting as a warehouse and transporter of many endo- and exogenous compounds. Shamsi et al. [3] investigated Huperzine A (HpzA), a natural sesquiterpene alkaloid (*Huperzia serrata)* used in various neurological conditions, including Alzheimer’s disease (AD), binding to HSA using advanced computational approaches such as molecular docking and molecular dynamic (MD) simulation, followed by fluorescence-based binding assays. The binding of HpzA with HSA showed an appreciable binding affinity and many intermolecular interactions. MD trajectory analyses (i.e., RMSD, RMSF, *R_g_*, SASA, and hydrogen bonding) suggested that the HSA–HpzA docked complex was quite stable with minimal conformational alterations. Fluorescence-based binding ascertained the actual binding affinity between HpzA and HSA, suggesting that HpzA binds to HSA with a significant affinity. The results provide a basis for setting up an experimental platform and information regarding the mechanism of HpzA interactions with has.

Simultaneous administration of two or more drugs might lead to a competition between the two drugs to bind to a similar site present on the serum albumin. The interaction between erlotinib (ERL) and bovine serum albumin (BSA) was studied in the presence of quercetin (QUR), a flavonoid with antioxidant properties, by Wani et al. [4]. Quercetin may interfere with the binding of the other concomitantly used drugs, impacting their pharmacokinetics and displacing them from their albumin binding sites, leading to higher free drug fractions of ERL in the system in the presence of QUR. Such interference may lead to a toxic or a subtherapeutic response of the drug in the presence of QUR. The presence of quercetin in the BSA-ERL system reduces the binding constant of the BSA-ERL system to almost half of what was observed in its absence. Thus, co-administration of QUR and ERL might influence the pharmacokinetics of ERL and needs to be investigated by in vivo studies.

Globally, hepatitis E accounts for an estimated mortality rate of –3.3% in the infected population. HEV has a genome of –7.2 kb plus-stranded RNA with a 5′-methylguanine (m^7^G) cap accorded by guanylyltransferase (GTase) and methyltransferase (MTase). RNA capping is essential for the viruses’ evasion of the host immune system and to produce other viral proteins by protecting the viral mRNA from nucleases. In the case of HEV, the 5′ m^7^G cap has been demonstrated to play an additional role, increasing infectivity in non-human primates and cultured hepatoma cells. Despite being an important enzyme, few studies have examined the functional and structural aspects of the HEV MTase. In the present study, Hooda et al. [5] cloned, expressed, and purified MTase spanning 33–353 amino acids of HEV genotype 1. The activity of the purified enzyme and the conformational changes were established through biochemical and biophysical studies. This study suggested an indispensable role of Mg^2+^ in MTase activity and stability. This work also established the optimal experimental conditions helpful for the screening of inhibitor libraries against HEV MTase to identify potential inhibitors.

The inhibition of glycation has also been shown to be beneficial in the treatment of diabetic complications. The medicinal properties of garlic (*Allium sativum*) are well documented and were further investigated by Khan et al. [6] as a natural plant phytochemical that can prevent glycation with fewer adverse side effects. Biophysical, biochemical, and molecular docking investigations were conducted to assess the antiglycating, antioxidant, and protein structural protection activities of garlic. Garlic extract exhibited significant levels of protection in protein structural stabilization against glycation. This research significantly supports the numerous therapeutic properties of garlic and addresses the question of the interdependence of various biological activities and their antioxidant capacity. The authors also suggested to include garlic into any existing preventative or treatment approach for glycation-induced health complications in diabetic patients.

Quetiapine (QTP) is a second-generation (short-acting atypical) antipsychotic drug of dibenzothiazepine (class) used to treat schizophrenia, acute bipolar disorder, and major depression in adolescents and adults. In their study, Zargar et al. [7] investigated the binding of QTP to human serum albumin (HSA). The QTP–HSA binding interactions showed moderate binding affinity of QTP toward HSA and involved hydrogen bonding and hydrophobic interactions. The also showed the complex formation between QTP and HSA and a static quenching mechanism. The QTP binding region at subdomain IB of HSA was investigated. This study is expected to help understand the drug’s mechanisms and pharmacokinetics for further clinical research and novel drug delivery systems.

Donor–acceptor complexation plays an important role, especially in the field of biochemical energy transfer processes. The formation of brilliantly colored CT complexes that absorb visible light is frequently linked to charge transfer interactions between electron acceptors and donors. In biological systems, mechanisms requiring molecular complexation and structural recognition include drug design, enzyme catalysis, and ion exchanges via lipophilic membranes. Alamri et al. [8] investigated a typical antipsychotic drug, Haloperidol’s (HPL), solid charge transfer (CT) products with 7,7,8,8-tetracyanoquinodimethane (TCNQ) and picric acid (PA). The findings of the study suggest that [(HPL)(TCNQ)] coupled with serotonin and dopamine more efficiently than HPL alone. In addition, [(HPL)(TCNQ)]–dopamine had a higher binding energy value than HPL–dopamine. The molecular dynamic simulation at 100 ns demonstrated that the [(HPL)(TCNQ)]–dopamine complex had a more stable interaction with the dopamine receptor than the HPL–dopamine complex.

Khan et al. [9] investigated the binding of caffeic acid and coumaric acid with α-amylase and analyzed the effect of these compounds on the formation of advanced glycation end-products (AGEs). Caffeic and coumaric acid bind with α-amylase and also inhibit the formation of AGEs to a certain extent. Caffeic acid possesses more inhibitory activity, which could be due to its planarity and hydrogen bonding potential. Van der Waals and hydrogen bonding are the major forces in polyphenols–protein interactions. Molecular docking, along with fluorescence quenching and synchronous fluorescence, displayed the ability of phenolics to form stable complexes with amylase. Moreover, these phenolics decrease AGE formation by inhibiting fructosamine. Furthermore, the oxidation of proteins boosted the effect of glycation; caffeic and coumaric acid, on the other hand, attenuate it by protecting thiol and carbonyl groups. The authors suggest more research on similar structures along with in vivo studies to design inhibitors for diabetic complications.

Belal et al. [10] implemented the repurposing of various FDA-approved ophthalmic medications for targeting MMP-2 and MMP-9 to establish a treatment outline for keratoconus (KC), a primary cause of corneal ectasia. Although the exact etiology and pathogenic mechanism are unknown, environmental and genetic variables are considered to play a role in the disease’s progression. The authors selected a group of thirty-two FDA-approved drugs and subjected them to virtual screening through docking against MMP-2 and MMP-9 proteins to identify the most promising inhibitors as a proposed computational mechanism to treat KC. The results indicate that some drugs may have potential activities against these proteins, opening the field to further biological studies. The docking results showed the ability of atenolol and ampicillin to accommodate well into the active sites of MMP-2 and MMP-9, respectively. Molecular dynamic simulations and MM-GBSA calculations point to the stability of the binding of both drugs to the respective enzyme, thus adding to the potential of both compounds in KC management. These encouraging findings pave the way for additional clinical investigations to confirm such theoretical findings.

Zhang et al. [11] employed HPLC-MS and network pharmacology to identify the active components and key signaling pathways of DGS. Transgenic zebrafish and HUVECs cell assays were used to evaluate the effectiveness of DGS. A total of 37 potentially active compounds were identified that interacted with 112 potential targets of CAD. Furthermore, PI3K-Akt, MAPK, relaxin, VEGF, and other signal pathways were determined to be the most promising DGS-mediated pathways. The findings of the study suggest that DGS may exert proangiogenic and vasodilatory effects through the activation of the VEGF/VEGFR2/Akt/Erk/eNOS signaling pathway. Molecular docking and molecular dynamics suggest that salvianolic acid C may be a key component in exerting angiogenic and vasodilatory effects.

NIMA-related kinase7 (NEK7) plays a multifunctional role in cell division and NLRP3 inflammasone activation. Any mutation or atypical expression of NEK7 leads to the development of cellular oncogenesis and may provoke a fatal inflammatory response, causing the tumorigenesis of multiple organs. These findings lend testimony to the involvement of NEK7 in the progression and development of numerous deadly diseases.

NEK7 is a promising therapeutic target for preventing and treating NEK7-related diseases. A few medications have recently been developed to target the NEK7-mediated inflammasome pathway, but the mechanism and treatment outcomes are not specific and consistent. Aziz et al. [12] focused on the virtual screening of 1200 benzene sulphonamide derivatives retrieved from the PubChem database by selecting and docking validation of the crystal structure of NEK7 protein (PDB ID: 2WQN). The compounds library was subjected to virtual screening using Auto Dock Vina. The binding energies of screened compounds were compared to standard dabrafenib. This study utilized deep learning models for the prediction of binding affinity, pIC_50_, and ADMET properties. Among the studied compounds, 762 showed good binding affinity and demonstrated a promising ADMET profile. Molecular dynamics simulations determined the protein–ligand complex was stable.

The increased incidence of tuberculosis cases and drug-resistant strains prompted researchers to identify novel drug targets and natural therapeutics with lesser toxicity. Therefore, three medicinal plants, *Achyranthes aspera* (*A. aspera*), *Calotropis gigantea* (*C. gigantea*), and *Calotropis procera* (*C. procera*), were investigated by Beg et al. [13] for their potential therapeutic intervention in Tuberculosis. The plants’ extracts were tested against different mycobacterial strains. *A. aspera* aerial and *C. gigantea* flower ash were found to be active against the *M. tuberculosis* H_37_Rv ATCC 27294 strains with an MIC value of 64 mg/L. A multitarget assessment study was used to identify the possible mycobacterial target proteins. Ten proteins, viz., *BpoC*, *RipA*, *MazF4*, *RipD*, *TB15.3*, *VapC15*, *VapC20*, *VapC21*, *TB31.7*, and *MazF9*, were found in the intersection of two categories, viz., available PDB dataset proteins and proteins classified in virulence, detoxification, and adaptation. In silico characterization identified *TB15.3* (Rv1636) in the intersection of the PPI network, which are the universal stress proteins. The MD simulation was used to determine the stability and accuracy of the complex. The results showed that the complex of *β*-amyrin and *Rv1636* was a stable complex, and the protein did not undergo unfolding during the simulation run. This study established a significant bridge in the field of mycobacterial biology in the targeting of Rv1636, a universal stress protein of mycobacteria, through natural phytoconstituents.
